# Investigating the associations of macular edema in retinitis pigmentosa

**DOI:** 10.1038/s41598-023-41464-z

**Published:** 2023-08-30

**Authors:** Juan D. Arias, Fritz Gerald P. Kalaw, Varsha Alex, Shaden H. Yassin, Henry Ferreyra, Evan Walker, Naomi E. Wagner, Shyamanga Borooah

**Affiliations:** 1 Jacobs Retina Center, University of California San Diego, San Diego, CA USA; 2 Ophthalmology – Retina Division, Shiley Eye Institute, The Viterbi Family Department of Ophthalmology, University of California San Diego, 9415 Campus Point Drive, San Diego, CA 92093 USA

**Keywords:** Medical research, Eye diseases, Retinal diseases

## Abstract

Macular edema (ME), the accumulation of intraretinal fluid in the macula, is a common sight affecting sequelae of retinitis pigmentosa (RP). However, it is unclear why some patients develop ME, and others do not. This study aims to identify associations between clinical-genetic factors in RP with ME. Patients with clinically confirmed RP cases were identified from the inherited retinal disease database at a large tertiary referral academic center. Demographic and genetic testing findings were noted. Additionally, optical coherence tomography volume scans were graded using a validated grading system. One hundred and six patients (73.1%) were found to have ME in at least one eye (OD = 88, mean = 37.9%, OS = 98, mean = 31.7%). Structurally, the presence of epiretinal membrane (ERM) (*p* < 0.007) and vitreo-macular traction (VMT) (*p* < 0.003) were significantly associated with ME. Additionally, X-linked (*p* < 0.032) and autosomal dominant inheritance (*p* < 0.039) demonstrated a significant association with ME, with *RP1* (*p* < 0.045) and *EYS* (*p* < 0.017) pathogenic variants also significantly associated with ME. This study, in a large cohort of RP patients, confirms previous retinal structural associations for ME in RP and identifies potential new genetic associations.

## Introduction

Macular edema (ME) is often the end result of a number of pathological degenerative processes or inflammation^1^. It is most frequently a consequence of hyper-permeable retinal blood vessels which cause extraversion of fluid and macromolecules into the retinal interstitium^2^. ME has been associated with numerous pathological ophthalmic diseases including, but not limited to, branch or central retinal vein occlusion (BRVO/CRVO)^3^, age-related macular degeneration (AMD)^4^, intraocular surgery complications^2^ and retinitis pigmentosa (RP)^5^. The increasing use of spectral-domain optical coherence tomography (SD-OCT) in clinical practice has facilitated the diagnosis of ME^6^. ME can have debilitating visual consequence, reducing central vision in patients who already have retinal dysfunction and who have already lost peripheral vision^5^. It is still currently unclear why ME occurs in RP. Various hypotheses have been suggested including disruption of the blood retina barrier, vitreo-macular traction, inflammatory disease, and iodine dysregulation^7^.

RP consists of a group of inherited diseases with a similar phenotype^8^. More than 60 genes are known to cause RP with over 3000 pathogenic variants^9^. The prevalence of ME in RP commonly seen in literature ranges from 8 to 58%, where much of the difference in prevalence lies in varying diagnostic tools and techniques used. Previous studies^34^ have found associations between autosomal recessive genes and ME in RP, but this can fluctuate greatly due to demographic criteria. RP can be inherited in an autosomal-recessive (AR) (50–60% of cases), autosomal-dominant (AD) (30%-40% of cases), or X-linked manner (5–15% of cases)^10–13^. Increasingly, genetic testing, using next generation sequencing, is being utilized to identify the molecular cause of RP. This has increased the yield of molecularly confirmed RP cases in recent years^14^.

The aim of the present study is to leverage a large cohort of RP cases in which majority patients have had genetic testing, to better understand whether there are any clinical features or genetic associations of ME in RP, with a hypothesis that certain demographic, clinical and genetic features are associated with the presence of ME in RP.

## Methods

### Study subjects

Patients were identified from the inherited retinal disease (IRD) database at UC San Diego (UCSD). This database contained clinical information, imaging data and genetic testing results for all patients with suspected IRD seen by three attending retinal physicians at UCSD from June 2008 to July 2021. The diagnosis of RP was confirmed and included, but was not limited to, a history of progressive peripheral vision loss or nyctalopia, and ocular examination findings of RP including bone spicule pigmentation, disc pallor and attenuated vessels and genetic confirmation. A cross-sectional, retrospective review of the clinical, genetic, and imaging findings from the database, and medical records, was performed. Eyes were included for the morphometric analysis if they had SD-OCT imaging. Visual acuity was tested utilizing a Snellen eye chart and Intraocular Pressure (mmHg) was obtained with an iCare tonometer. Values were taken independently for each eye and were not averaged. For consistency, patient’s clinical data was obtained from their initial visit only to the UCSD department of ophthalmology using either electronic medical record or physical medical charts at which stage patients were treatment naïve.

### Morphological and quantitative grading

All the eyes were imaged using Heidelberg Spectralis® SD-OCT (Heidelberg Engineering, Inc., Heidelberg, Germany) using a 49-section raster scan with an ART setting of 16. Two retinal fellows (VA, FK) graded images for the presence of ME, epiretinal membrane (ERM), posterior vitreous detachment (PVD) and vitreo-macular traction (VMT). ERM is a fibro cellular membrane on the inner aspect of the retina, which often develops with age and through fibrosis. It is not initially usually vision threatening but can progress with time^15^. PVD occurs when the posterior vitreous pulls away from the inner limiting membrane of the retina^16^. A partial or incomplete PVD can result in VMT when the posterior vitreous does not completely detach from the macula leading to anatomic disruption of the fovea^17^.

Differences in grading were resolved by a retinal attending (SB). ME was graded when at least one b-scan showed the presence of hypo-reflective spaces between the ganglion cell layer/ nerve fiber layer to the ellipsoid zone (EZ) (See Supplementary Fig. S1). ERM was graded on the presence of a hyper-reflective band on the internal limiting membrane (ILM), with or without inner retinal layer fibrillations or disorganization. PVD, was graded when a thin hyper-reflective band within the vitreous was not seen attached to any layer of the internal limiting membrane or retinal vasculature following review of all the b scans in the volume scan. Lastly, VMT was graded depending on the presence of thin or thick hyper-reflective band on the ILM with notable evagination of foveal contour. Foveal thickness was measured using the Heidelberg Spectralis® SD-OCT generated Foveal Thickness Maps upon scan analysis. The grading system was validated using inter-grader validation and test–retest validation.

### Genetic variables

Genetic testing was performed in clinic using various Clinical Laboratory Improvement Amendments (CLIA) certified labs using either blood or saliva samples and the results were interpreted by a clinical genetic counselor. Next-generation sequencing (NGS), exome sequencing, and/or targeted Sanger sequencing were the primary genetic testing approaches.

Genetic results were presented using the recommendations of the American College of Medical Genetics and Genomics (ACMG)^18^ and included “uncertain significance”, “likely benign”, “benign”, “likely pathogenic” or “pathogenic” variants^18^. Variants of uncertain significance, likely benign or benign nature were excluded from the analysis. Patients with confirmed genetic testing but no morphological data, were also included into the genetic analysis.

### Statistical analysis

A Generalized Linear Mixed Effects Model (GLMM)(See Supplementary Table S1) was used to predict the binary presence of ME and association with genes in order to account for the use of two eyes from subjects. Each analyzed eye was independent from the other. Random intercepts were used to account for within-subject variability, due to the correlated nature of our data.

The top genes were analyzed. These genes were chosen based on the proportion of ME presence, not by sample size. Pearson’s Chi Squared and Fisher’s Exact Test provided indications for which genes held a relationship with ME.

The statistical significance of differences in continuous and categorical patient level characteristics between groups were assessed with two-sample t-tests and Fisher’s Exact Test. Some variables, with two-factors or multifactor levels, were assessed using Fisher’s Test and Pearson’s Chi-Squared Test. For eye level characteristics, statistically significant differences were assessed using generalized linear mixed-effects models with random intercepts to account for within-subject variability. We fitted a GLMM using the binomial presence of ME as the dependent variable, controlling for a mixture of eye level and patient level characteristics as the dependent variables. The GLMM was appropriate for our data due to the intercorrelated nature of subject fellow eyes. Non-proportionate data was determined by the statistician based on the sample size and parameters; smaller, non-parametric subsets were found to be unproportionate and called for the use of Fisher’s exact test. Parametric data was noted by the statistician to be normally distributed and if the mean, rather than the median, accurately represented the center of distribution. The statistical analysis was performed using R statistical programming software (R Version 4.2.0). An alpha level of *p* < 0.05 was considered statistically significant for all tests and was used as the threshold to reject a null hypothesis.

### Ethics approval

Patient data was masked, and consenting was practiced in accordance with institutional policies set within the department. This research and all methodologies used adhered to the tenets of the Declaration of Helsinki. The study was approved by the Institutional Review Board of the University of California, San Diego (UCSD). The mentioned committee is a part of the UCSD Human Research Protections program.

### Informed consent

Informed consent to release health information was also obtained for every patient prior to their first appointment at UCSD. All patients signed an informed consent and patients over the age of 18 signed on their own behalf or had a legal guardian present if they were minors. An informed consent was obtained from the parent or guardian if the patient was a minor and below the age of 18. All patients or guardians were able to read and write in the language in which the consent was presented to them.

## Results

### Demographic findings

The database included 571 patients with suspected IRD, with 170 (29.8%) RP patients. Due to the retrospective nature of this study, 25 patients and three eyes had missing SD-OCT imaging which resulted in their exclusion from imaging analysis. The final number of RP patients included in the analysis was 287 eyes from 145 patients (Table 1).

**Table 1:** Patient level and eye-level characteristics.

	Total subjects = 145 Eyes = 287	ME present subjects = 106*	ME not present subjects = 39	*p* value
Gender (%)				
Male	68 (46.9%)	50 (47.2%)	18 (46.2%)	0.508
Female	75 (51.7%)	54 (50.9%)	21 (53.8%)	
Transgender	1 (0.7%)	1 (0.9%)	0 (0%)	
Ethnicity (%)				
White	64 (44.1%)	43 (40.6%)	21 (53.8%)	0.878
Hispanic	29 (20.0%)	20 (18.9%)	9 (23.1%)	
Asian	15 (10.3%)	14 (13.2%)	1 (2.6%)	
Middle Eastern	15 (10.3%)	11 (10.4%)	4 (10.3%)	
Black	4 (2.8%)	4 (3.8%)	0 (0%)	
(Other)	18 (12.4%)	14 (13.2%)	4 (10.3%)	
Thickness (µm) (95% CI)	274.07 (256.83, 291.31)	237.80 (226.87, 248.72)	367.63 (324.22, 411.05)	0.001
VA (BCVA) (95% CI)	5.72 (4.78, 6.67)	5.67 (4.53, 6.81)	5.87 (4.14, 7.60)	0.653
IOP (mmHg) (95% CI)	13.78 (12.70, 14.85)	13.06 (11.97, 14.15)	15.72 (13.04, 18.40)	0.177

*VA Visual acuity, *IOP* Intraocular pressure.

The mean age of our analyzed cohort was 49.73 (SD = 19.75) years of age. A sub-analysis was performed by comparing the prevalence of ME, PVD/VMT/ERM in a younger and older age group; however, no significant associations were noted. Even though no significant associations were found, ME was more prevalent in the younger group (49.73 years >) (68.9%) compared to the older group (60.3%).

Chi-Squared and Fisher’s Exact Test were used to analyze differences in the proportion of subjects with ME present between ethnic groups. Asian ME proportions (n = 14, mean = 13.2%) versus Caucasian ME proportions (n = 43, mean = 40.6%) (Table 2) found that the true proportions between these groups were not the same (*p* = 0.026). Caucasian ethnicities had the lowest prevalence of ME in RP (Table 2).

**Table 2:** Eye-level Asian ME proportions versus Caucasian ME proportion.

	ME	No ME	Proportion with ME	*p* value
Asian	24	4	0.828	0.026
Caucasian	75	53	0.586	

No significant differences were found between any other groups. An odds ratio of 1.44 (*p* = 0.621) was found for the presence of ME in males, although an association of ME with gender failed to meet statistical significance during modeling.

### Clinical and inheritance findings

A detailed validation study was performed to see if there was agreement between two independent retinal specialists for the grading of ME, ERM, PVD, and VMT. Inter-grader testing found an agreement of 92.5% with a Cohens Kappa of 0.850 (*p* value > 0.001). Overall, the test–retest of both graders combined had an agreement of 88.80%, which translated to a Cohen’s Kappa score of 0.77 and *p* value < 0.001, signaling substantial agreement and thus validating the grading method.

Of the 287 eyes from 145 patients, 186 eyes (73.1%) from 106 patients presented with ME (OD = 88, mean = 37.9%, OS = 98, mean = 31.7%). Other pathologies such as ERM, PVD, and VMT were also frequently identified (Table 4). The senior grader was only involved in confirming grading in 176 individual scans (15.3%) where there was no agreement between grading retinal specialists in at least one measure.

A Pearson’s Chi-Squared Test analyzed normally distributed and parametric clinical features and inheritance types with ME in RP. It was found that intraretinal fluid (*p* < 0.005) and foveal thickness (*p* < 0.001) were both significantly associated with ME (Table 3). Better visual acuity was also associated with less ME (*p* < 0.05) (Table 3). This further validated the grading system as ME would usually result in increased foveal thickness, have intraretinal fluid and would likely be associated with lower vision. Interestingly, AD variants (n = 18, mean = 6.3%, *p* = 0.032) (Table 3) showed a significantly increased prevalence of having ME.

**Table 3:** Final model independent variables to measure associations with ME.

Covariate	Odds ratio	*p* value
Age	0.99	0.819
Male	1.44	0.621
Thickness	0.97	< 0.001
Intraretinal fluid	0.01	0.005
Visual acuity	0.89	0.05
Autosomal dominant	0.01	0.032

Utilizing Fisher’s test for analysis for morphological and inheritance types with a non-parametric nature, an association was found between ERM presence (*p* <  = 0.007) (mean = 12.4%) and ME (Table 4). Additionally, patients with VMT (*p* = 0.005) showed a significant association with ME (mean = 4.9%) (Table 4).

**Table 4:** Relationship between ME and Fishers exact test results on unproportionate variables.

	Distribution	*p* value
Post-cataract surgery		
Not present	230 (80.7%)	0.641
Present	55 (19.3%)	
IRF		
Not present	258 (90.5%)	< 0.001
Present	27 (9.5%)	
Pallor		
Not present	245 (86.0%)	0.289
Present	40 (14.0%)	
ERM		
Not present	234 (87.6%)	0.007
Present	33 (12.4%)	
VMT		
Not present	254 (95.1%)	0.005
Present	13 (4.9%)	
X-linked recessive		
Not present	267 (93.7%)	0.039
Present	18 (6.3%)	

*Two-hundred thirty eyes did not undergo cataract surgery.

Meanwhile analyzing the associations with types of genetic inheritance, X-linked inheritance (n = 18, mean = 6.3%, *p* = 0.039) (Table 4) was also found to have a significant association with ME presence. It should be noted that the majority of patients (230 eyes) did not have cataract surgery when first examined. No association with prior cataract surgery and ME was found. There were no other significant findings regarding other clinical or morphological variables.

### Genetic findings

**Figure 1:**
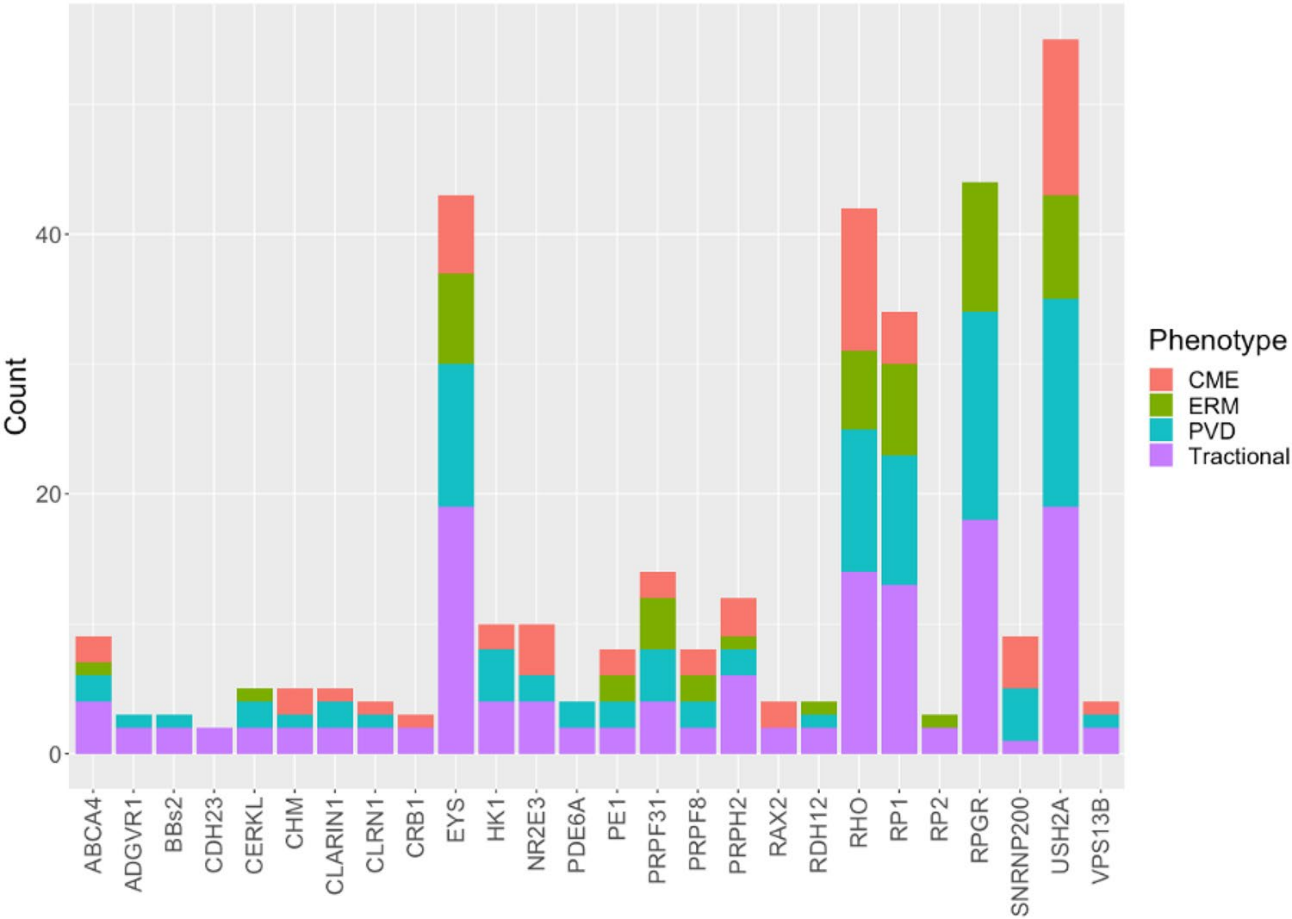
Genotypic–phenotypic descriptive plot (Count is per eye).

Having identified that certain types of inheritance were associated with ME, we investigated the genetic associations further to see if any genes were associated with ME. One-hundred and five patients had genetically confirmed RP. Fifty-eight of those patients presented with ME. A generalized linear mixed effect model was used to analyze the most prevalent genes noted to have ME (38 eyes): *RHO* (*n* = 5), *USH2A* (*n* = 8), *RP1* (*n* = 10), and *EYS* (*n* = 14) (Table 5). Looking at individual genes, only *RP1* (*p* < 0.045) and *EYS* (*p* < 0.017) (Table 5) were found to have a statistically significant association with ME in our cohort therefore suggesting a possible genetic predisposition for ME in RP. Figure 1 depicts the phenotypic-genotypic relationship between the morphological variables and individual genes found in our cohort. See Supplementary Table S2 for a complete genotypic glossary of the cohort.

**Table 5:** Generalized linear mixed effect model to assess individual gene association with ME.

ME	RHO	USH2A	RP1	EYS
Not present	9	5	2	2
Present	5	8	10	15
Proportion with ME	35.71%	61.54%	83.33%	88.20%
Coefficient	1.73	2.69	5.93	6.83
*p* value	0.279	0.224	0.045	0.017

*Inf Infinite.

**Coefficient value of this table demonstrates each gene’s association strength with ME presence, with a smaller coefficient indicating a lower spread of data relative to the mean.

## Discussion

In the present study, we hypothesized that the presence of ME in RP was associated with demographic, clinical or genetic findings in our cohort of RP patients. To validate the findings, we reviewed previous papers in the area and noted that results of the relationship between ME-RP clinical abnormalities of this cohort, were similar with those of previous studies^19^. Visual acuity had an inverse relationship with the presence of ME. Patients with increased foveal thickness tended to have worse vision, while those with a reduced foveal thickness tended to have better vision. However, this finding has not been the case for all studies regarding ME-RP^20,21^. Kim et al. did not believe that there was a link between the two; rather, ME had a leading role in disrupting foveal regions which could severely impact visual acuity^21^. Their study analyzed 128 RP patients (220 eyes), which included 30 patients (46 eyes) with ME. They defined ME to be intraretinal cystoid like spaces at the central region of the macula^21^. Our findings supplement mentioned existing literature supporting a deterioration of vision with increased ME.

Regarding ethnicity, Caucasian subjects represented majority of our cases. Unsurprisingly, Caucasians were also those with the highest absolute number of ME. Asian subjects were the only group found to have a higher prevalence (85.7%) compared to Caucasian individuals (58.6%). To our knowledge a similar ethnic comparison has not been performed previously. Interestingly, this finding contrasts with a study of Asian patients from Japan in which a cohort of 323 Japanese subjects had only an 8% prevalence of ME in RP^22^. The stark differences between this study results with ours may be explained by the fact that many of our patients were primarily referred for retinal abnormalities such as ME and RP was discovered later upon a clinical examination. SD-OCT was used in the ascribed study to detect the presence of ME, which was categorized by cystoid like spaces or intraretinal fluid^22^. In our cohort, the origin of Asian subjects was not differentiated, and this may account for the differences seen. There is a dearth of previous literature focusing on ethnic differences in ME in RP and could benefit from further future study.

ERM and VMT were the two prominent structural variables associated with ME. Both are common distortions seen in ME, regardless of RP. A high count of vitreomacular interface disorders (VMID), which include ERM and VMT have previously been linked to RP^23^. ERM was the most prevalent disorder in our cohort, followed by VMT. These findings were supported by the findings of Fragiotta et al. which analyzed 145 RP (257 eyes) cases and had a cohort of predominantly Caucasian patients^23^. This study did not include ME presence, instead they sought to specifically explore the prevalence ERM, PVD and macular holes and report their progression over a longitudinal time frame^23^. Other studies have included ME and have similarly concluded that the prevalence of VMID’s in ME-RP is high, again validating the findings in our cohort^24–26^. However, only one recently published study has accounted for genetic variables when analyzing VMIDs in RP^27^. Marques et al. similarly identified ERM as the most common VMID in RP. This study focused on genetically confirmed cases of syndromic vs non-syndromic RP and the prevalence it may have with ME and VMID; however, possible genetic associations with ME were not explored^27^. In regard to treatment involving VMIDs, it should be noted that surgical intervention can carry greater risk in treatment and carbonic anhydrase inhibitors are often the mainstay form of treatment due to its safe use and proven benefit^4,5^.

Regarding the genetic findings of our study, 26 genes were included in our analyses. *EYS* (12.5%) and *RP1* (16.67%) were the only genes found to be significantly associated with ME in our cohort. Although *RPGR* was the most common X-linked mutation, it was not found to be significantly associated with ME in RP. However, X-linked inheritance still shared an association with ME despite the high *p* value of *RPGR* due to the other X-linked genes found in our cohort.

*EYS*’s function is believed to be a facilitator for protein transport between the inner and outer segments of the photoreceptors^28^. *RP1* has been identified as a facilitator of protein transport in the photoreceptor and maintaining cilial structure^28^. *RP1* is expressed in the outer segments of rod and cone photoreceptors as well as other tissues in the human body^29^. However, ME did not seem to be exclusive to transport or ciliary proteins in our cohort. As a result, although a genetic association was identified a genetic causal mechanism for ME is not currently clear.

Researchers have previously hypothesized several mechanisms for ME in RP including loss of Müller cells leading to loss of protection against ME^5,30^. Other theories, such as an inflammatory mechanism, are plausible considering ME can be improved by steroids in a subset of patients^5,31^. The findings of associations with inheritance pattern and especially association with genes do not appear to have been reported in previous studies and would be of interest to study in larger cohorts. Considering that UCSD is a tertiary referral center, this could contribute to this and sway the results found in our study. The use of latanoprost was not accounted for in our cohort and could also be useful in future clinical studies.

The limitations of the present study include its retrospective nature, with some patients excluded due to missing imaging. Additionally, various forms of genetic testing were used. Initially, most tests were for single gene testing with later focused on exome panels. As a result, molecular causes of RP are likely to have been under reported. A limitation of any genetic study is that even with current exome sequencing the molecular cause of IRDs is only identified in approximately 60–70% of cases and so we will still be limited in finding a genetic association with features of RP^32,33^.

Although many casual genes were identified, sample sizes for each individual gene could be considered relatively small and should be counted as a limitation. The associations found regarding RP1 could be influenced by family effects being a confounding factor. A total of 7 families with 2 members each were a part of our cohort and could be considered a confounding factor. In addition, types of ME were not noted in this study, and are the subject of ongoing work by several groups and a relatively simple grading criteria was used. This warrants further study once definitions have been agreed upon. Lastly, the lack of angiography testing prevents the differentiation of macular edema being the result of vascular disease versus maculopathies such as VMT and ERM. Future prospective studies involving angiography and genetic sampling could prove beneficial.

In conclusion, this present study confirms the retinal structural associations identified in previous studies and adds to the literature regarding associations of ME in RP. In addition, the present study suggests genetic associations with ME, although the replication of these genetic findings using cohorts from other centers would be useful to strengthen the findings in our study.

### Supplementary Information

Supplementary Information 1.

Supplementary Information 2.

## Data Availability

Data used in this study is not available for public use due to privacy protection protocols. This information can be retrieved from the corresponding author upon request.
