# Supplementary material for: Investigating the associations of macular edema in retinitis pigmentosa
Source: Sci Rep. 2023 Aug 30;13:14187. doi: 10.1038/s41598-023-41464-z (PMC10469217; doi:10.1038/s41598-023-41464-z)
Supplement: Supplementary file 1 — Supplementary Information 1. [file 41598_2023_41464_MOESM1_ESM.docx]

**Legend**

Table S1. Fitting Generalized Linear Mixed Effects Models

Fig S1. Representative example of macular edema in a 17-year-old male

Table S2. Genotypic Glossary
