# Supplementary material for: Investigating the associations of macular edema in retinitis pigmentosa
Source: Sci Rep. 2023 Aug 30;13:14187. doi: 10.1038/s41598-023-41464-z (PMC10469217; doi:10.1038/s41598-023-41464-z)
Supplement: Supplementary file 2 — Supplementary Information 2. [file 41598_2023_41464_MOESM2_ESM.docx]

| Final GLMM Model | Intercept Coefficient | p-value | Marginal R² | Conditional R² |
| --- | --- | --- | --- | --- |
| ME ~ Age + Sex + Thickness + IRF + VA + AD-Inheritance + E(ID) | 12.01 | <0.001 | 0.641 | 0.883 |

**Supplementary Table S1: Fitting Generalized Linear Mixed Effects Model**

^*^ME = Macular Edema; IRF = Intraretinal Fluid; VA = Visual Acuity; AD = Autosomal Dominant

Note: E(ID)* represents the random intercept to account for within-subject variability


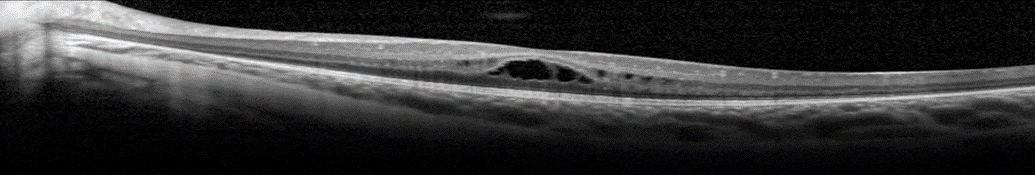
**Supplementary Figure S1: Representative example of macular edema in a 17-year-old male**

**Supplementary Table S2: Genotypic Glossary**

| **Gene** | **Segregation (recessive and X-linked variants)** | **Variant** | **Variant Classification*** | **Reference** | **Variant Type** | **REVEL** (listed for missense)** | **SpliceAI*** (listed for intronic and splice variants)** |
| --- | --- | --- | --- | --- | --- | --- | --- |
| RHO |  | c.888G>C (p.Lys296Asn) | Likely pathogenic | PMID:1765377 | Missense | Deleterious (Supporting) (0.67) |  |
| EYS | Homozygous | c.6192-1G>A | Likely pathogenic | N/A | Null (splice site) |  | Splice-Altering (0.98) |
| SNRNP200 |  | c.2042G>A (p.Arg681His) | Pathogenic | PMID: 21618346 | Missense | Deleterious (Strong) (0.94) |  |
| RDH12 | Homozygous | c.295C>A (p.Leu99Ile) | Pathogenic | PMID:15322982 | Missense | Deleterious (Supporting) (0.68) |  |
| USH2A | Compound heterozygous | c.2299del (p.Glu767SerfsTer21) | Pathogenic | PMID:9624053 | Null (frameshift) |  |  |
|  |  | c.2276G>T (p.Cys759Phe) | Pathogenic | PMID:10729113 | Missense | Deleterious (Moderate) (0.9) |  |
| PRPF31 |  | c.18_24delinsCCCCTG (p.Glu6AspfsTer59) | Likely pathogenic | N/A | Null (frameshift) |  |  |
| RP1 |  | c.1625C>G (p.Ser542Ter) | Pathogenic | PMID:22917891 | Null (nonsense) |  |  |
| CDH23 | Unknown phase | c.1446_1447insTT (p.Leu483PhefsTer36) | Pathogenic | PMID: 21940737 | Null (frameshift) |  |  |
|  |  | c.227_233dup (p.Phe78LeufsTer8) | Pathogenic | PMID: 21940737 | Null (frameshift) |  |  |
| FLVCR1 | Homozygous | c.1092+5G>A | Pathogenic | PMID:27353947 | Intronic |  | Splice-Altering (low) (0.36) |
| EYS | Homozygous | c.5928-2A>G | Pathogenic | PMID:20333770 | Null (splice site) |  | Splice-Altering (0.99) |
| PRPF31 |  | c.912_914dupTGT (p.Val305dup) | Likely pathogenic | N/A | Other (duplication) |  |  |
| RHO |  | c.541G>A (p.Glu181Lys) | Pathogenic | PMID:1833777 | Missense | Deleterious (Moderate) (0.85) |  |
| ABCA4 | Unknown phase | c.4469G>A (p.Cys1490Tyr) | Pathogenic | PMID:9973280 | Missense | Deleterious (Strong) (0.95) |  |
|  |  | c.2744-1G>T | Likely pathogenic | PMID:26780318 | Null (splice site) |  | Splice-Altering (0.99) |
| USH2A | Unknown phase | c.1606T>C (p.Cys536Arg) | Pathogenic | PMID:10909849 | Missense | Deleterious (Moderate) (0.87) |  |
|  |  | c.12152_12153insTT (p.Glu4051AspfsTer2) | Pathogenic | PMID:10729113 | Null (frameshift) |  |  |
| SNRNP200 |  | c.3260C>T (p.Ser1087Leu) | Pathogenic | PMID:19878916 | Missense | Deleterious (Supporting) (0.67) |  |
| PRPH2 |  | c.394del (p.Gln132LysfsTer7) | Pathogenic | PMID:24608669 | Null (frameshift) |  |  |
| PCDH15 | Homozygous | c.-29+1G>C | Likely pathogenic | N/A | Null (splice site) |  | Splice-Altering (0.95) |
| RPGR | Hemizygous | c.353A>C (p.Gln118Pro) | Likely pathogenic | N/A | Missense | Deleterious (Supporting) (0.75) |  |
| EYS | Unknown phase | c.9036del (p.Leu3013SerfsTer6) | Pathogenic | PMID: 27735924 | Null (frameshift) |  |  |
|  |  | c.(?_-538-1)_(-448+1_-447-1)del | Pathogenic | N/A | Null (deletion) |  |  |
| USH2A | Unknown phase | c.2299del (p.Glu767SerfsTer21) | Pathogenic | PMID:9624053 | Null (frameshift) |  |  |
|  |  | c.14893_14900del (p.Val4965ArgfsTer33) | Likely pathogenic | N/A | Null (frameshift) |  |  |
| CHM | Hemizygous | c.715C>T (p.Arg239Ter) | Pathogenic | PMID:16087855 | Null (nonsense) |  |  |
| PRPF8 |  | c.6910T>G (p.Phe2304Val) | Pathogenic |  | Missense | Deleterious (Strong) (0.97) |  |
| PRPF8 |  | c.6910T>G (p.Phe2304Val) | Pathogenic |  | Missense | Deleterious (Strong) (0.97) |  |
| EYS | Unknown phase | c.2652dup (p.Lys885Ter) | Likely pathogenic | N/A | Null (nonsense) |  |  |
|  |  | c.1068C>A (p.Cys356Ter) | Likely pathogenic | N/A | Null (nonsense) |  |  |
| ABCA4 | Unknown phase | c.6383A>G (p.His2128Arg) | Likely pathogenic | PMID:10206579 | Missense | Deleterious (Strong) (0.97) |  |
|  |  | c.3G>T (p.Met1?) | Pathogenic | N/A | Null (disruption of initiator codon) |  |  |
| RP2 | Hemizygous | c.234dup (p.Ala79CysfsTer6) | Likely pathogenic | N/A | Null (frameshift) |  |  |
| CLRN1 | Homozygous | c.144T>G ( p.Asn48Lys) | Pathogenic | PMID:12080385 | Missense | Uncertain (0.52) |  |
| ABCA4 | Unknown phase | c.4793C>A (p.Ala1598Asp) | Pathogenic | PMID:10958761 | Missense | Deleterious (Supporting) (0.69) |  |
|  |  | exon 7 deletion (chr1:94.548.898-94.549.008) | Pathogenic | N/A | Null (deletion) |  |  |
| RP1 | Unknown phase | c.2042delinsAA (p.Ile681LysfsTer17) | Pathogenic | PMID:11527933 | Null (frameshift) |  |  |
|  |  | c.597C>G (p.Tyr199Ter) | Pathogenic | PMID:24265693 | Null (nonsense) |  |  |
| CNGB1 | Unknown phase | c.3139_3142dup, p.(Ala1048GlyfsTer13) | Pathogenic | PMID:28056120 | Null (frameshift) |  |  |
|  |  | c.2957A>T (p.Asn986Ile) | Pathogenic | PMID:28056120 | Missense | Deleterious (Supporting) (0.7) |  |
| USH2A | Unknown phase | c.2276G>T (p.Cys759Phe) | Pathogenic | PMID:10729113 | Missense | Deleterious (Moderate) (0.9) |  |
|  |  | c.918_919insGCTG (p.Ser307AlafsTer17) | Likely pathogenic | PMID:22004887 | Null (frameshift) |  |  |
| EYS | Homozygous | c.6191+1G>A | Pathogenic | PMID:29159838 | Null (splice site) |  | Splice-Altering (0.96) |
| EYS | Homozygous | c.6191+1G>A | Pathogenic | PMID:29159838 | Null (splice site) |  | Splice-Altering (0.96) |
| EYS | Unknown phase | c.6571+1G>A | Pathogenic | PMID:18836446 | Null (splice site) |  | Splice-Altering (0.99) |
|  |  | c.1111C>T (p.Gln371Ter) | Likely pathogenic | N/A | Null (nonsense) |  |  |
| EYS | Unknown phase | c.6416G>A (p.Cys2139Tyr) | Pathogenic | PMID:20237254 | Missense | Deleterious (Supporting) (0.76) |  |
|  |  | c.8107G>T (p.Glu2703Ter) | Pathogenic | PMID:24618324 | Null (nonsense) |  |  |
| RPGR | Hemizygous | c.493G>C (p.Gly165Arg) | Likely pathogenic | N/A | Missense | Deleterious (Strong) (0.98) |  |
| AHI1 | Compound heterozygous | c.2623+2T>A | Likely pathogenic | PMID:16453322 | Null (splice site) |  | Splice-Altering (0.93) |
|  |  | Deletion (Exons 20-22) | Likely pathogenic | N/A | Null (deletion) |  |  |
| RHO |  | c.563G>A (p.Gly188Glu) | Pathogenic | PMID:8317502 | Missense | Deleterious (Supporting) (0.71) |  |
| RHO |  | c.8G>A (p.Gly3Asp) | Likely pathogenic | PMID:28559085 | Missense | Deleterious (Supporting) (0.73) |  |
| RP1 |  | c.2285_2289del (p.Leu762TyrfsTer17) | Pathogenic | PMID:10391211 | Null (frameshift) |  |  |
| NR2E3 | Homozygous | c.119-2A>C | Pathogenic | PMID:15459973 | Null (splice site) |  | Splice-Altering (0.95) |
| USH2A | Unknown phase | c.2299del (p.Glu767SerfsTer21) | Pathogenic | PMID:9624053 | Null (frameshift) |  |  |
|  |  | c.920_923dup (p.His308GlnfsTer16) | Pathogenic | PMID:10729113 | Null (frameshift) |  |  |
| USH2A | Homozygous | c.6967C>T (p.Arg2323Ter) | Pathogenic | PMID:25649381 | Null (nonsense) |  |  |
| HK1 |  | c.2539G>A (p.Glu847Lys) | Pathogenic | PMID:25316723 | Missense | Deleterious (Moderate) (0.82) |  |
| HK1 |  | c.2539G>A (p.Glu847Lys) | Pathogenic | PMID:25316723 | Missense | Deleterious (Moderate) (0.82) |  |
| USH2A | Unknown phase | c.2276G>T (p.Cys759Phe) | Pathogenic | PMID:10729113 | Missense | Deleterious (Moderate) (0.9) |  |
|  |  | c.(12294+1_12295-1)_(14133+1_14134-1)del (deletion encompassing exons 63-64) | Pathogenic | PMID:26969326 | Null (deletion) |  |  |
| EYS | Unknown phase | c.2620C>T (p.Gln874Ter) | Pathogenic | PMID:25491159 | Null (nonsense) |  |  |
|  |  | g.(65986130_67624031inv) rearrangement | Pathogenic | N/A | Other (inversion) |  |  |
| CERKL | Homozygous | c.847C>T (p.Arg283Ter) | Pathogenic | PMID:14681825 | Null (nonsense) |  |  |
| RHO |  | c.68C>A (p.Pro23His) | Pathogenic | PMID:2137202 | Missense | Deleterious (Strong) (0.98) |  |
| RHO |  | c.180C>G (p.Tyr60Ter) | Pathogenic | PMID:24265693 | Null (nonsense) |  |  |
| EYS |  | c.8133_8137del (p.Phe2712CysfsTer33) | Pathogenic | PMID:20333770 | Null (frameshift) |  |  |
|  |  | c.1123G>T (p.Glu375Ter) | Pathogenic | N/A | Null (nonsense) |  |  |
| ABCA4 | Unknown phase | c.3522+1G>T | Likely pathogenic | N/A | Null (splice site) |  | Splice-Altering (0.97) |
|  |  | c.5714+5G>A | Pathogenic | PMID:9666097 | Intronic |  | Splice-Altering (low) (0.31) |
| PRPH2 |  | c.629C>T (p.Pro210Leu) | Pathogenic | PMID:11485765 | Missense | Deleterious (Moderate) (0.9) |  |
| NR2E3 | Compound heterozygous | c.226C>T (p.Arg76Trp) | Pathogenic | PMID:10655056 | Missense | Uncertain (0.37) |  |
|  |  | c.227G>A (p.Arg76Gln) | Pathogenic | PMID:10655056 | Missense | Uncertain (0.38) |  |
| RPGR | Heterozygous in female | c.269_270del (p.Lys90IlefsTer25) | Pathogenic | N/A | Null (frameshift) |  |  |
| RPGR | Hemizygous | c.2405_2406del (p.Glu802GlyfsTer32) | Pathogenic | PMID:18552978 | Null (frameshift) |  |  |
| CLRN1 | Homozygous | c.144T>G (p.Asn48Lys) | Pathogenic | PMID:12080385 | Missense | Uncertain (0.52) |  |
| RPGR | Hemizygous | c.2706_2707del (p.Glu903GlyfsTer175) | Pathogenic | N/A | Null (frameshift) |  |  |
| RPGR | Hemizygous | c.2706_2707del (p.Glu903GlyfsTer175) | Pathogenic | N/A | Null (frameshift) |  |  |
| RPGR | Heterozygous in female | c.2706_2707del (p.Glu903GlyfsTer175) | Pathogenic | N/A | Null (frameshift) |  |  |
| RS1 | Hemizygous | c.1A>T (p.Met1?) | Pathogenic | PMID:17031297 | Null (disruption of initiator codon) |  |  |
| RPGR | Heterozygous in female | c.2706_2707del (p.Glu903GlyfsTer175) | Pathogenic | N/A | Null (frameshift) |  |  |
| RHO |  | c.541G>A (p.Glu181Lys) | Pathogenic | PMID:1833777 | Missense | Deleterious (Moderate) (0.85) |  |
| VPS13B | Compound heterozygous | Deletion of exons 4-13 | Pathogenic | N/A | Null (deletion) |  |  |
|  |  | c.11124C>A (p.Tyr3708Ter) | Pathogenic | N/A | Null (nonsense) |  |  |
| RPGR | Hemizygous | c.2405_2406del (p.Glu802GlyfsTer32) | Pathogenic | PMID:10480356 | Null (frameshift) |  |  |
| PDE6A | Unknown phase | c.475-2A>G | Likely pathogenic | N/A | Null (splice site) |  | Splice-Altering (0.99) |
|  |  | c.84C>A (p.Tyr28Ter) | Likely pathogenic | N/A | Null (nonsense) |  |  |
| ADGRV1 | Unknown phase | c.(7945+1_7946-1)_16611+1_16612-1)del (deletion encompassing exons 34-77) | Pathogenic | N/A | Null (deletion) |  |  |
|  |  | c.1477C>T (p.Arg493Ter) | Likely pathogenic | PMID:22147658 | Null (frameshift) |  |  |
| RPGR | Hemizygous | c.2236_2237 del (p.Glu746ArgfsTer23) | Pathogenic | PMID:10932196 | Null (frameshift) |  |  |
| RAX2 | Homozygous | Partial Deletion (Exon 2) | Pathogenic | N/A | Null (deletion) |  |  |
| BBS1 | Homozygous | c.1169T>G (p.Met390Arg) | Pathogenic | PMID:12118255 | Missense | Deleterious (Supporting) (0.66) |  |
| USH2A | Homozygous | c.2149T>A (p.Cys717Ser) | Pathogenic | N/A | Missense | Deleterious (Strong) (0.94) |  |
| ARL6 | Homozygous | c.362G>A (p.Arg121His) | Likely pathogenic | PMID:28130426 | Missense | Uncertain (0.55) |  |
| RHO |  | c.568G>A (p.Asp190Asn) | Pathogenic | PMID:1765377 | Missense | Uncertain (0.45) |  |
| PRPH2 |  | c.659G>C (p.Arg220Pro) | Pathogenic | PMID:29555955 | Missense | Deleterious (Moderate) (0.88) |  |
| RPGR | Hemizygous | c.3096_3097del (p.Glu1033ArgfsTer45) | Pathogenic | PMID:11857109 | Null (frameshift) |  |  |
| USH2A | Homozygous | c.956G>A (p.Cys319Tyr) | Likely pathogenic | PMID:10729113 | Missense | Uncertain (0.52) |  |
| USH2A | Homozygous | c.9799T>C (p.Cys3267Arg) | Pathogenic | PMID:17085681 | Missense | Deleterious (Moderate) (0.78) |  |
| RP1 |  | c.2200del (p.Ser734ValfsTer4) | Likely pathogenic | PMID:28076437 | Null (frameshift) |  |  |
| RP1 |  | c.2200del (p.Ser734ValfsTer4) | Likely pathogenic | PMID:28076437 | Null (frameshift) |  |  |
| RP1 |  | c.2029C>T (p.Arg677Ter) | Pathogenic | PMID:1783394 | Null (nonsense) |  |  |
| RP1 |  | c.2671del (p.Arg891GlyfsTer5) | Pathogenic | N/A | Null (frameshift) |  |  |

* Variant classification listed as reported by Clinical Laboratory Improvement Amendments (CLIA)-certified laboratory if American College of Medical Genetics and Genomics (ACMG) classification system was used. In cases where the laboratory did not report a classification, variants were evaluated for their pathogenicity using Franklin (https://franklin.genoox.com) using ACMG criteria.

**Variant classification listed as reported by Clinical Laboratory Improvement Amendments (CLIA)-certified laboratory if American College of Medical Genetics and Genomics (ACMG) classification system was used. In cases where the laboratory did not report a classification, variants were evaluated for their pathogenicity using Franklin (https://franklin.genoox.com) using ACMG criteria.

***SpliceAI (https://github.com/Illumina/SpliceAI) is a deep-learning tool used to evaluate predicted effect of splice variants. Scores range from 0-1, with higher scores reflecting greater liklihood of splicing alteration.
